# Cardiomyocyte­-specific expression of the nuclear matrix protein, CIZ1, stimulates production of mono-nucleated cells with an extended window of proliferation in the postnatal mouse heart

**DOI:** 10.1242/bio.021550

**Published:** 2016-12-01

**Authors:** Sumia A. Bageghni, Georgia A. Frentzou, Mark J. Drinkhill, William Mansfield, Dawn Coverley, Justin F. X. Ainscough

**Affiliations:** 1 LICAMM, University of Leeds, Leeds LS2 9JT, UK; 2 Stem Cell Institute, University of Cambridge, Cambridge CB2 1QR, UK; 3 Biology Department, University of York, York YO10 5DD, UK

**Keywords:** Cardiac function, Cardiomyocyte, CIZ1, DNA replication, Nuclear-matrix protein

## Abstract

Myocardial injury in mammals leads to heart failure through pathological cardiac remodelling that includes hypertrophy, fibrosis and ventricular dilatation. Central to this is inability of the mammalian cardiomyocyte to self-renew due to entering a quiescent state after birth. Modulation of the cardiomyocyte cell-cycle after injury is therefore a target mechanism to limit damage and potentiate repair and regeneration. Here, we show that cardiomyocyte-specific over-expression of the nuclear-matrix­-associated DNA replication protein, CIZ1, extends their window of proliferation during cardiac development, delaying onset of terminal differentiation without compromising function. CIZ1-expressing hearts are enlarged, but the cardiomyocytes are smaller with an overall increase in number, correlating with increased DNA replication after birth and retention of an increased proportion of mono-nucleated cardiomyocytes into adulthood. Furthermore, these CIZ1 induced changes in the heart reduce the impact of myocardial injury, identifying CIZ1 as a putative therapeutic target for cardiac repair.

## Abstract (precis)

**Summary:** An inducible mouse model was developed to show that CIZ1 extends the window of cardiomyocyte proliferation and reduces the impact of injury on cardiac function.

## INTRODUCTION

Heart disease is a leading cause of death in the developed world, with major efforts aimed towards repairing the heart after injury. A key issue that compromises cardiac repair is resistance to regeneration of differentiated mammalian myocardium. Thus, any significant insult that induces cardiomyocyte (CM) death results in irreparable damage. This is exacerbated by remodelling events that drive cardiac fibrosis, development of arrhythmias, and heart failure. Increasing effort has been directed towards understanding molecular processes that underlie CM development, to unlock ‘hidden’ regeneration potential. Greater understanding of mechanisms that control renewal could facilitate translation into therapeutic application. Central to this is the resistance of the adult CMs to re-enter the cell cycle following terminal differentiation (Pasumarthi and Field, 2002). In humans the majority of CMs are mono-nucleated, some bi-nucleated and a very small proportion multi-nucleated (Olivetti et al., 1996). In contrast, mouse CMs undergo nuclear division without cytokinesis shortly after birth, so the majority become suspended in a bi-nucleated state in the adult heart. Stresses such as hypoxia induce tissue remodelling through CM-mediated activation of resident non-myocytes (NMs) resulting in deposition of extracellular matrix and formation of scar tissue that lacks key properties of healthy myocardium (Turner, 2011; Frentzou et al., 2015). Strategies for regenerating damaged myocardium include stem cell therapies, such as bone marrow, embryonic and induced pluripotent stem cells (reviewed in Ainscough et al., 2012). Methods have also been reported for direct reprogramming of cardiac fibroblasts into immature CMs (Ieda et al., 2010; Qian et al., 2013). However, these approaches are not yet sufficiently advanced to enable correct integration of appropriately differentiated cells into local resident myocardium, with clinical trials showing limited improvement (Behfar et al., 2014; Broughton and Sussman, 2016). The potential for resident CMs to re-enter the cell cycle and proliferate to repair damage is an attractive prospect. Unlike mammals, lower vertebrates are able to achieve this, involving an initial phase of de-differentiation around the damaged zone, and subsequent proliferation of nearby healthy CMs (Jopling et al., 2010). The neonatal mouse heart retains similar repair capacity, but this is lost as the cells bi-nucleate within the first weeks after birth (Porrello et al., 2011). Indeed, bi-nucleated cells in adult myocardium are terminally quiescent, whereas the small proportion of mono-nucleated cells retain proliferation potential (Bersell et al., 2009). Thus, a viable alternative approach to stem cell therapy might be manipulation of regulatory mechanisms to shift the CM population towards the mono-nucleated form. In mammalian cells G1 progression is regulated by cyclin D/CDK4/6 and cyclin E/CDK2 (Sherr and Roberts, 2004), while S-phase entry is promoted by cyclin E/CDK2 and cyclin A/CDK2 (Coverley et al., 2002). The nuclear matrix protein CDKN1A-interacting zinc finger protein-1 (CIZ1) plays a pivotal role in co-ordinating activity of these molecules to promote initiation of DNA replication, by targeting them to specific sub-nuclear sites (Coverley et al., 2005; Ainscough et al., 2007; Copeland et al., 2010, 2015). Here, we use a new conditional transgenic mouse model to demonstrate that CIZ1 can promote CM proliferation and enhance formation of mono-nuclear cells, without compromising cardiac function. The data also suggest that CIZ1 is a candidate therapeutic target for reducing the impact of injury on myocardial function.

## RESULTS

### Differential expression of CIZ1 in CM and NM populations of the heart

**Fig. 1.**
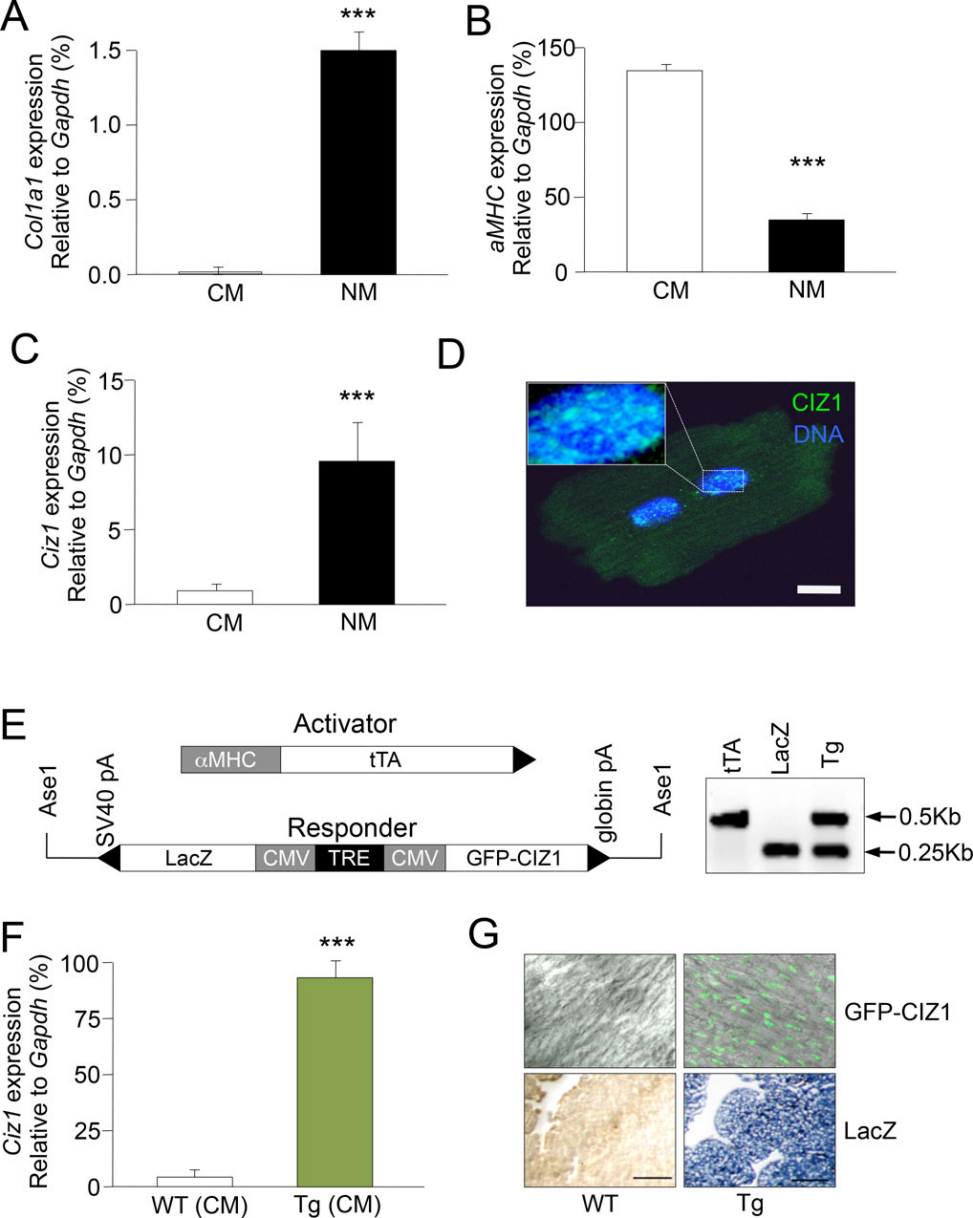
**Expression analysis of CIZ1 in WT and Tg CMs.** (A) Determination of cardiomyocyte (CM) fraction purity by qRT-PCR. *Col1a1* was expressed exclusively in non-myocytes (NM). (B) *αMHC* was primarily restricted to CMs. *αMHC* in NMs suggests presence of CMs co-sedimenting with NMs. (C) *Ciz1* was expressed primarily in NMs. (D) CIZ1 protein is localised to nuclear speckles in adult WT CMs, detected with antibody 1793. Scale bar=10 μm. (E) Schematic representation of activator and responder transgenes, with PCR assay for positive identification. Tg mice were positive for both transgenes. (F) Relative *Ciz1* expression in isolated CMs of WT and Tg mice. (G) Nuclear localisation of ectopic CIZ1 was demonstrated by GFP fluorescence (green) in fresh sections of 3-week-old Tg hearts, confirmed by β-gal protein staining (blue). Scale bar=100 μm. *n*=3-4 mice/group. Data represented as mean±s.e.m. ***P≤0.001 by two-tailed *t*-test.

Purified CMs were isolated from mouse hearts by a modified Langendorff perfusion method (Frentzou et al., 2015). Purity was confirmed by lack of expression of the NM marker *Col1a1* (Fig. 1A). Although some CMs were also present in the NM fraction, detected by expression of *aMHC* (Fig. 1B), qRT-PCR showed that ∼90% of *Ciz1* mRNA in the heart was NM-derived, comprising fibroblast, endothelial, smooth muscle and inflammatory cells (Fig. 1C). All of these cell types retain ‘proliferation potential’, unlike the terminally differentiated CM population in which CIZ1 expression was approximately 20-fold less. However, at the protein level CIZ1 foci were readily observed in CM nuclei, suggesting long-term retention of ‘old’ CIZ1 in this cell type after differentiation, with no further role in promoting replication initiation (Fig. 1D).

### Conditional CIZ1 mouse model

To test whether renewed production of CIZ1 in CMs could prolong cell cycle activity, we produced a novel transgenic mouse in which expression of new CIZ1 protein could be induced. The model utilized two transgenes, one encoding transactivator and the other encoding a responsive LacZ/GFP-CIZ1 reporter (Fig. 1E). CM-specific transactivator expression was achieved through the aMHC promoter, which drives expression in atrial CMs during embryogenesis, and is activated in ventricular CMs at birth and throughout adulthood. The CM-specific transactivator molecule drives *LacZ/GFP-Ciz1* expression by binding the tetracycline response element (TRE). We previously employed the same transactivator line to drive specific expression of *LacZ/AT1R* in cardiomyocytes, and reported clear cell type specificity through extensive analysis of LacZ reporter expression in a range of tissues and developmental stages (Ainscough et al., 2009). Thus, LacZ and GFP-CIZ1 were only expressed in CMs of double transgenic mice (Tg). From three transgenic founders we selected two lines for further characterization (see Materials and Methods). Line CIZ24 expressed CM-specific CIZ1 at approximately 100-fold higher than endogenous levels (Fig. 1F), comparable to the level seen in adult testis (Greaves et al., 2012). Thus, although this level of CIZ1 exceeds that in most other tissues and developmental stages, it remains within physiologically relevant levels. As with endogenous CIZ1, GFP-CIZ1 protein accumulated in CM nuclei (Fig. 1G). This new model provided a unique opportunity to address the influence of enhanced CIZ1 on cell cycle progression specifically in CMs, and to assess its impact on cardiac function.

### CIZ1-expressing hearts are enlarged but not functionally impaired

**Fig. 2.**
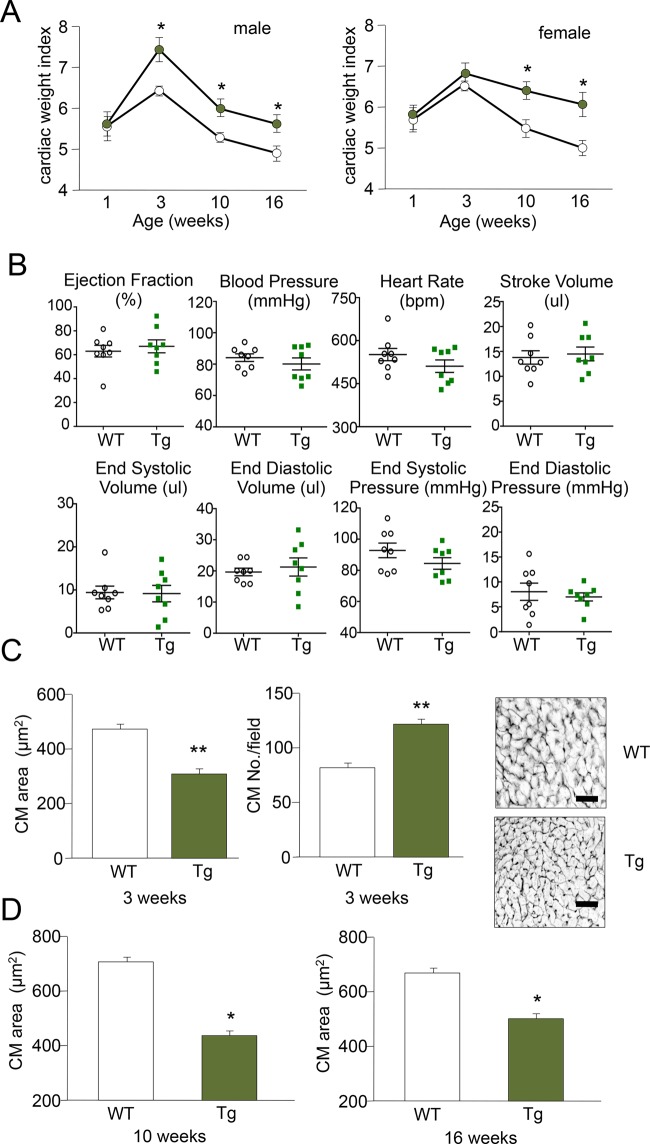
**Impact of CIZ1 on cardiac structure and function.** (A) Cardiac weight index of WT (white) and Tg (green) males and females from neonate to young adulthood. (*n*=8-17 mice/group). (B) Millar catheter assessment of cardiac function at 16 weeks showed no difference between groups. (C) Ventricular CM cross-sectional area and number/field, determined using the membrane stain WGA and calculated using ImageJ (NIH) (*n*=7-10 images/mouse, three mice/group). (D) Differential cross sectional area was maintained at 10-16 weeks. Scale bar=50 μm. Data represented as mean±s.e.m. *P≤0.05, **P≤0.01 by two-tailed *t*-test.

CIZ24 mice were examined for evidence of cardiac disorder at 1, 3, 10, and 16-weeks after birth (Fig. 2A). Neonatal Tg hearts were not significantly larger, but enlargement became evident and sustained at later stages in both males and females. Millar catheter assessment at 16-weeks found no evidence that the hearts were functionally compromised, with no significant differences in end systolic or diastolic volumes, ejection fraction or stroke volume (Fig. 2B, Table 1). Importantly, this showed that continued production of CIZ1 in mature CMs is not detrimental to heart function. Consistent with this, histological assessment demonstrated that enlargement was not associated with cellular hypertrophy, fibrosis or dilatation. Instead Tg CMs were significantly smaller in cross section (Fig. 2C,D), indicating that the total CM number in the adult heart was greater than that in control hearts.

**Table 1.**
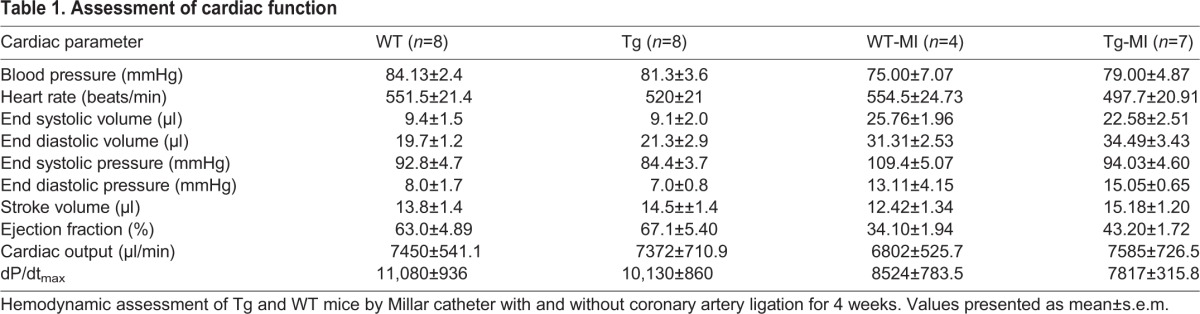
**Assessment of cardiac function**

### CIZ1-expressing hearts have more mono-nucleated CMs

**Fig. 3.**
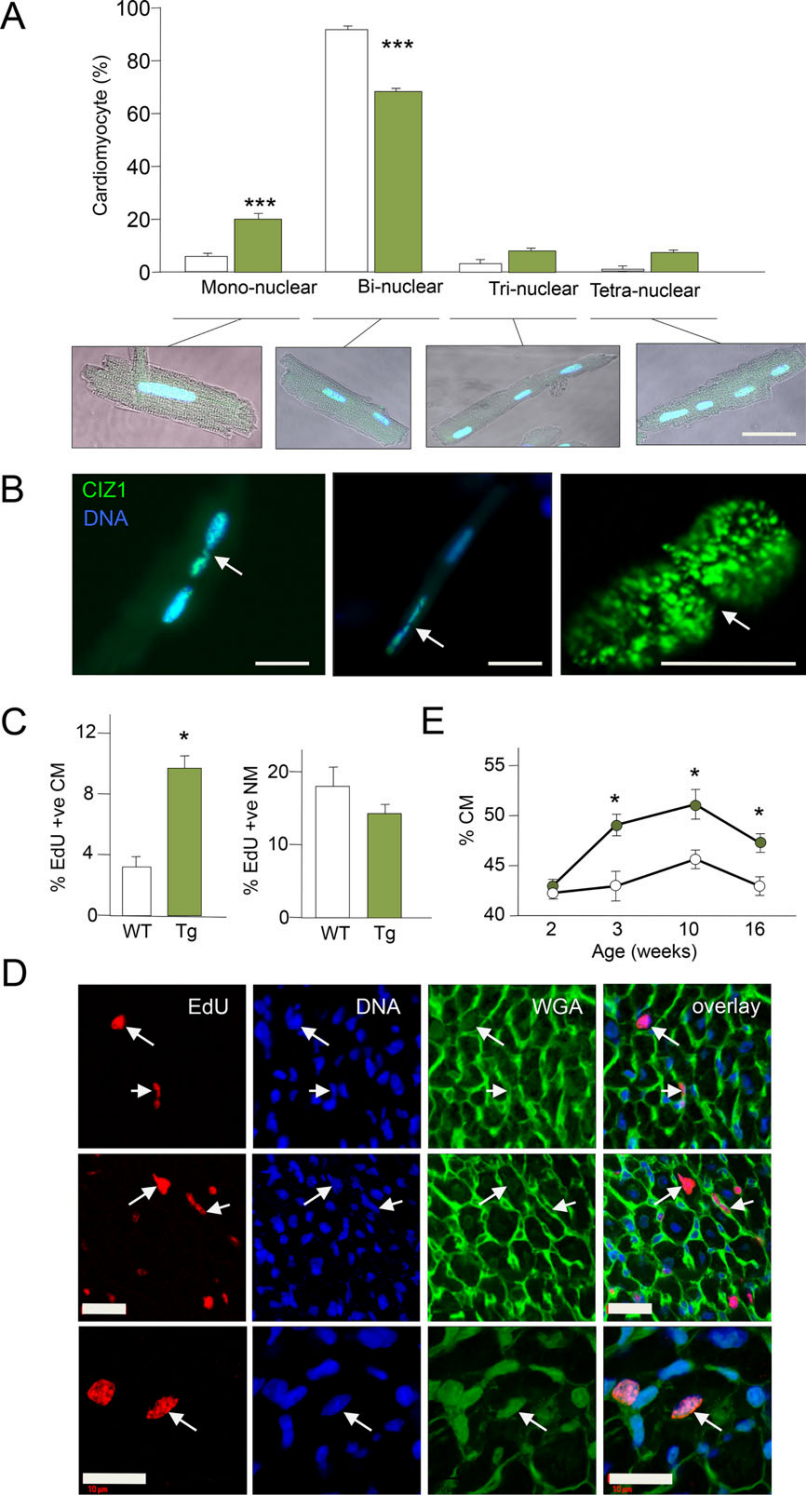
**CIZ1 promotes CM mono-nucleation and DNA replication.** (A) Proportion of mono-, bi-, tri- and tetra-nucleated CMs in WT (white) and Tg (green) hearts at 12 weeks (*n*=>500 CM/mouse, four mice/group), with representative examples of GFP-positive (green) Tg nuclei. (B) Examples of partially divided GFP-positive nuclei (arrows). (C) Hearts of 2-week-old Tg mice had threefold more replicating CMs than WT, detected following incorporation of EdU for 48 h (*n*=10-12 sections/mouse, >1000 nuclei, three mice/group). A slight decrease was observed in replicating NMs. (D) Examples of replicating Tg NMs (short arrow) and CMs (long arrow). Cell identity was based on nuclear size/position relative to WGA-stained membrane (Frentzou et al., 2015). (E) CM:NM ratio during development from neonate to young adulthood, in WT (white) and Tg (green) mice. Scale bar=20 μm. Data represented as mean±s.e.m. *P≤0.05, ***P≤0.001 by two-tailed *t*-test.

CMs were isolated from hearts at 12 weeks and individual nuclei counted (Fig. 3A). The proportion of bi-nucleated CMs was significantly reduced in Tg hearts (69.5% from 88%), whereas the mono-nucleated population was significantly increased (20.15% from 6.5%). A small increase in number of multi-nucleated CMs was also noted. It was difficult to assign some cells into distinct groups as the nuclei were partially divided (Fig. 3B). Together, the results (smaller cells, larger heart, increase in mono-nuclear CMs) suggest increased cell cycle activity and proliferation after birth.

### An extended window for CM self-renewal

Using stringent criteria for positive identification of CMs in tissue sections (Frentzou et al., 2015), *in vivo* EdU incorporation at 3 weeks showed that the number of replicating CM nuclei was small in both wild-type (WT) and Tg hearts. To determine when cell cycle activity declines, 2-week-old animals were subjected to EdU incorporation for 48 h. The number of CMs undergoing DNA synthesis was significantly higher in Tg hearts, while the proportion of replicating NMs was reduced (Fig. 3C,D). This correlated with a sustained increase in proportion of CMs in Tg hearts (Fig. 3E). Thus, the window of CM proliferation is extended in CIZ1-expressing hearts.

### CIZ1 reduces the impact of injury on cardiac function

**Fig. 4.**
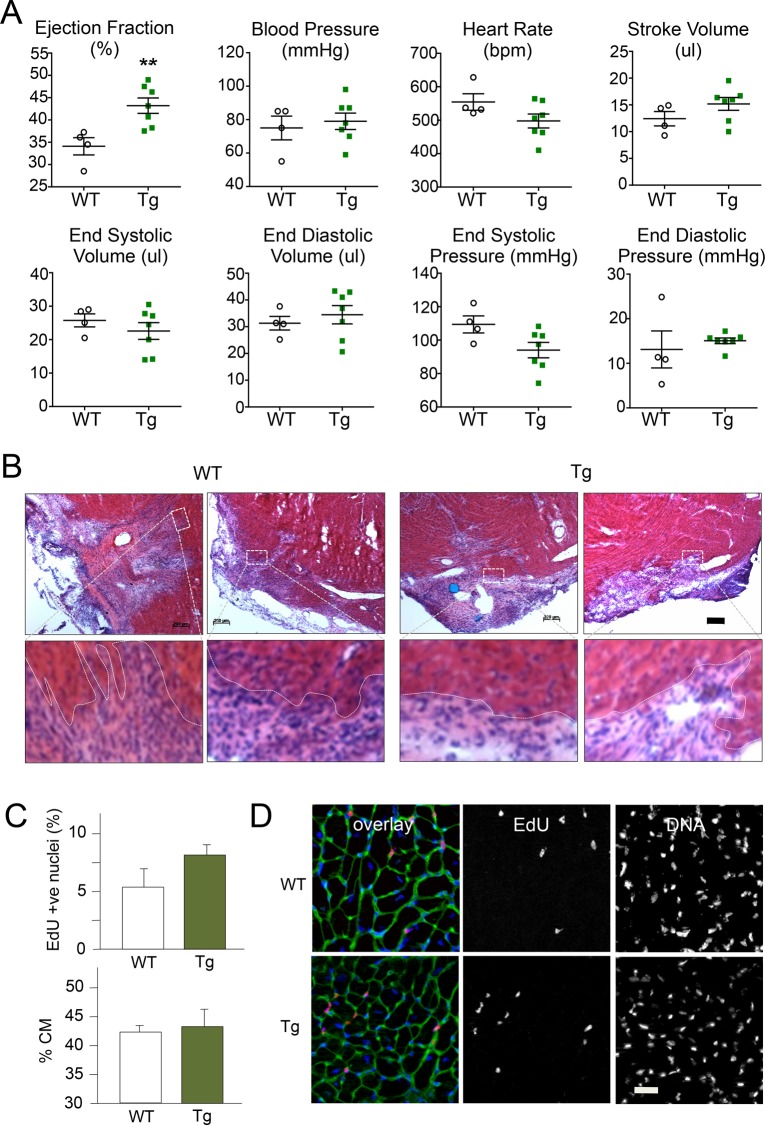
**CIZ1 reduces the impact of injury to the heart.** (A) 16-week-old WT and Tg mice were subjected to coronary artery ligation. At 20 weeks ejection fraction was significantly better in Tg mice. (B) Representative haematoxylin and eosin-stained sections of ligated hearts at 20 weeks. Magnified images show border zone between healthy (pink) and damaged myocardium, which appears blue due to increased NM density. Border zone is depicted by the white dotted line. Scale bar=200 µm. (C) Hearts of ligated Tg mice at 20 weeks exhibited marginally increased replication index, while CM:NM ratio was not different (*n*=613-940 nuclei, 7-10 images/mouse, three mice/group). (D) Representative images of EdU-positive nuclei (red) in WT and Tg hearts. Nuclei, blue; cell membrane, green. Scale bar=20 μm. Data represented as mean± s.e.m.

16-week-old mice were subjected to coronary artery ligation and left ventricular pressure-volume measurements compared to unligated controls after 4 weeks (Fig. 4A, Table 1). Although cardiac function was adversely affected in both groups, Tg animals were less affected, exhibiting 36% reduction in ejection fraction (67%-43%) compared to 54% reduction (63%-34%) in the WT animals. This correlated with increased end systolic and diastolic volumes indicative of cardiac dilatation, most exaggerated in WT hearts (Table 1). Representative hearts sectioned across the ligature showed no notable differences with both WT and Tg hearts exhibiting localised fibrosis with NM infiltration (Fig. 4B). 48 h prior to culling, three mice from each group were injected with EdU to enable assessment of DNA replication. Although the ratio of CM:NM in healthy myocardium appeared similar between groups, the overall replication index was slightly higher in Tg animals (Fig. 4C,D), albeit confined to cell nuclei with characteristic NM appearance. Thus, we observed no direct evidence for generation of new CMs in Tg hearts after injury, suggesting that the lessened impact of injury might be directly related to the increased number of smaller mono-nucleated CMs in the Tg heart.

## DISCUSSION

Together, the data demonstrate that CIZ1 can modulate cell cycle activity after birth and reduce the impact of injury to the heart. This is consistent with previous evidence that suggested a role for CIZ1 in DNA replication (Coverley et al., 2005), via interaction with cell cycle regulators (Mitsui et al., 1999; den Hollander and Kumar, 2006; Copeland et al., 2010, 2015). Our findings indicate that CIZ1 could be an important target for assisted regeneration, provided potential benefits outweigh possible risks. Although a range of alternatively spliced isoforms are expressed during embryonic development, adult tissues primarily express full-length *Ciz1* (Greaves et al., 2012). Some variants have been associated with a range of proliferative disorders including cancers, suggesting that deregulation of normal CIZ1 function might negatively impact on identity (Warder and Keherly, 2003; den Hollander et al., 2006; Rahman et al., 2007; Higgins et al., 2012). However, complete lack of CIZ1 has also been shown to promote tumorigenesis (Nishibe et al., 2013), suggesting tumour suppressor properties. Although there is no direct evidence to implicate full-length CIZ1 in any disease state the available evidence does suggest that caution is warranted. Given that expression of CIZ1 is tightly regulated during development (Greaves et al., 2012), and deviation from normal has potential to promote tumorigenesis, care must be taken in any attempt to manipulate CIZ1 for therapeutic gain.

A number of investigations have previously reported reactivation of the CM cell cycle through direct manipulation of other regulators. These include cyclins D1-3 (Soonpaa and Field, 1997; Soonpaa et al., 1997; Pasumarthi and Field, 2002; Zhu et al., 2009), cyclin A2 (Chaudhry et al., 2004; Cheng et al., 2007) CDK2 (Liao et al., 2001), p27 KIP1 (Poolman et al., 1999), p53 and p193 (Nakajima et al., 2004). Up-regulation of cyclin D1 promoted multi-nucleation, while CDK2 shifted the balance towards mono-nucleation (Soonpaa et al., 1997; Liao et al., 2001). Promisingly, cyclins A2 and D2 were reported to promote infarct regression (Pasumarthi et al., 2005; Cheng et al., 2007). Less promising, heart enlargement induced by CDK2 over-expression did not persist into adulthood, and the cell-cycle-modulating effect of p27 deletion was lost after 6 days (Poolman et al., 1999). For CIZ1 we found that the influence was comparatively stable, with mice exhibiting enlarged hearts even at 20 weeks. This might reflect the central role of CIZ1 in the DNA replication process, acting as a hub to bring together each of its interacting partners in a timely manner (Ainscough et al., 2007; Copeland et al., 2010). However, lack of direct evidence for continued DNA replication at later time points means this possibility requires further investigation.

In association with the increased number of small CMs a significant shift was observed in the balance from bi-nucleation towards mono-nucleation. This finding is important, as it was demonstrated previously that, in species which have myocardial regenerative potential, mono-nucleated CMs divide more successfully than bi-nucleated CMs (Matz et al., 1998). Over 90% of zebrafish CMs, which have a high regenerative capacity, are mono-nucleated (Wills et al., 2008). It has also been shown that differentiated mouse mono-nucleated CMs have regenerative potential, with capacity to disassemble their sarcomeres and undergo cell division (Bersell et al., 2009). It is not clear, therefore, why the mononucleated CMs in the human heart (majority population) are resistant to regeneration, suggesting additional as-yet-unexplained blocks to cell cycle reactivation are still to be discovered. Interestingly, knockout of the homeobox gene *Meis1* in mice promoted mono-nucleation (Mahmoud et al., 2013), suggesting that MEIS1 is one component that acts to restrain cell cycle re-entry. Our results show that CIZ1 promotes generation of mono-nucleated CMs and reduces the impact of injury on cardiac function in the adult. However, in isolation modulation of CIZ1 is not likely to be sufficient to induce cardiac regeneration and repair after injury. Further work to unlock the mechanisms that regulate cell-cycle inhibition in ageing cardiomyocytes may enable CIZ1 modulation to be a useful component of a toolbox for regenerative medicine.

## MATERIALS AND METHODS

### Animals

All experiments were performed with ethical approval from the University of Leeds under UK Home Office authorization. *GFPCiz1*/*LacZ*-Tg mice were generated by pronuclear injection of an inducible GFP full-length mouse *Ciz1* construct into CBA/C57BL6 fertilized eggs as described previously (Ainscough et al., 2009). Three positive lines were identified (CIZ12,15, 24) and copy number and integrity assessed by Southern blot. Each line was crossed with αMHC-tTA mice (FVB.Cg-Tg(Myh6-tTA)6Smbf/J; Jackson laboratories) to drive CM-specific expression. CIZ15/tTA mice did not express *Ciz1* or *LacZ.* CIZ12/tTA showed low-level *Ciz1* and mosaic *LacZ*, while CIZ24/tTA showed robust *Ciz1* expression and *LacZ* in all CMs. αMHC-tTA was detected using primer tTA1 (5′-CGCTGGGGGGCATTTTACTTTA-3′) with tTA2 (5′-CATGTCCAGATCGAAATCGTC-3′); *GFPCiz1/LacZ* using primer LacZ4 (5′-AATGGTCTGCTGCTGCTGAACG-3′) with LacZ5 (5′-GGCTTCATCCACCACATACAGG-3′).

### Cardiac function and myocardial injury

Myocardial infarction (MI) was induced by left coronary artery ligation at the atrial inferior border. Millar catheter analysis was performed 4 weeks later as described previously (Ainscough et al., 2009; Frentzou et al., 2015). A 1.4F miniature pressure-volume catheter (SPR-839, Millar Instruments) was inserted through the right carotid and ascending aorta into the left ventricle. Data was recorded using MPVS-300 (Millar Instruments), Chart5Pro (AD instruments) and PVAN 3.6 (Miller Instruments).

### Cardiac weight index

Excised hearts were washed in PBS, atria removed for *LacZ* assessment, and ventricles blotted and weighed. Samples not used immediately for RNA or histology were snap-frozen and stored at −80°C. Ventricle/body weight (mg/g) was recorded as cardiac weight index.

### Cardiac cell isolation and qRT-PCR

CMs and NMs were separated into fractions using a modified Langendorff apparatus as described (Frentzou et al., 2015). Following filtration to remove clumps, a CM pellet was settled by gravity, then washed in cold digestion buffer. For RNA, CMs were purified away from remaining NMs by resuspension and centrifugation in cold PBS (3×1 min) at 50 ***g***, then resuspended in TRI-reagent (Ambion). The NM supernatant from the CM settling was further cleared by sequential centrifugation (4×1 min). The remaining supernatant was centrifuged at 500 ***g*** (10 min). The resultant NM pellet was resuspended in TRI-reagent. Extracted RNAs were DNase treated, then reverse-transcribed with Superscript III (Invitrogen). qRT-PCR was performed using gene-specific primer-probes for *Ciz1*, *Col1α1*, *αMHC* and *Gapdh* (Table 2), on an ABI-7500-PCR system, normalised to *Gapdh.* Relative expression was calculated as 2^−ΔCT^×100 and presented as a percentage of *Gapdh* expression.

**Table 2.**
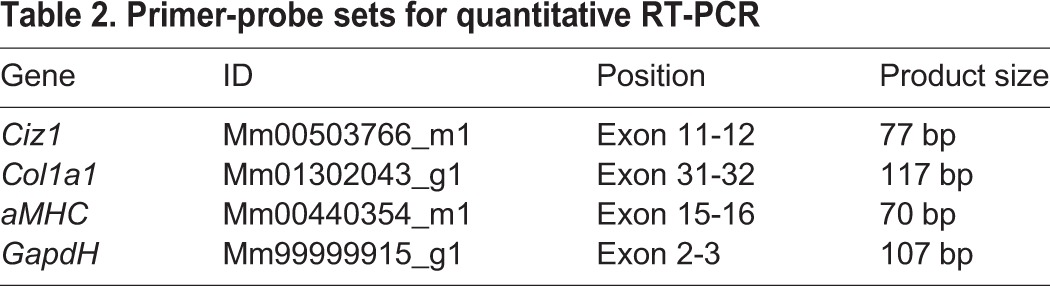
**Primer-probe sets for quantitative RT-PCR**

### DNA replication/EdU assay

Mice were injected once daily with 100 µg EdU in PBS, and hearts excised after 24-48 h. Click-iT EdU assay was carried out on 10 μm heart cryosections following manufacturer's instructions (Molecular Probes).

### Immunofluorescence

Isolated CMs were fixed in cold ethanol. Endogenous CIZ1 was detected using antibody-1793 (Coverley et al., 2005). Cell membranes and nuclei were visualised with wheat germ agglutinin (Vector Laboratories) and Hoechst 33258 (Sigma), respectively. Samples were mounted in VectorShield (Vector Laboratories). Images were taken using a Zeiss AxioImager Z.1 microscope and AxioVision software (Zeiss), then prepared using Adobe Photoshop.

### Statistical analysis

Data are expressed as mean±s.e.m., analysed by two-tailed *t*-test using GraphPad Prism5. *P≤0.05, **P≤0.01, ***P≤0.001.
